# Engaging the AQ10 to Predict Professional Burnout or Poor Work-Related Psychological Wellbeing Among Anglican Clergy in Wales

**DOI:** 10.1007/s10943-024-02006-7

**Published:** 2024-01-29

**Authors:** Leslie J. Francis, Alison B. Sailer, V. John Payne, Ursula McKenna

**Affiliations:** 1 Centre for Educational Development, Appraisal and Research (CEDAR), University of Warwick, Coventry, UK; 2 World Religions and Education Research Unit, Bishop Grosseteste University, Lincoln, UK; 3 College of Life and Health Sciences, Brunel University, London, UK; 4 Department of Psychology, Wrexham Glyndwr University, Wrexham, UK

**Keywords:** Clergy Studies, Burnout, Affective Disorders, Autism, Personality

## Abstract

The ten-item Autism Spectrum Quotient (AQ10) is a self-report instrument originally designed to identify referrals for professional diagnosis for Autism Spectrum Disorders (ASD). Recent studies suggest that this instrument may also be tapping more generalised affective disorders. Working with this interpretation, this study examines the predictive power of the AQ10 to account for additional variance, after personal and personality factors have been taken into account, on the two scales of the Francis Burnout Inventory. Data provided by 220 Anglican clergy serving in Wales demonstrated that 8.6% of the participants recorded six or more red flags on the AQ10 (and so qualified for referral for specialist diagnostic assessment) and that higher scores on the AQ10 are associated with significantly lower levels of satisfaction in ministry and with significantly higher levels of emotional exhaustion in ministry. These data suggest that screening with the AQ10 may be helpful in identifying clergy vulnerable to professional burnout and to poor work-related psychological wellbeing, in addition to its primary purpose of screening for ASD.

## Introduction

Recent years have seen a burgeoning interest in researching the correlates of individual differences in professional burnout among clergy in various denominations (see for example, Picornell-Gallar & González-Fraile, 2023). As yet this research field has not explored the connection between professional burnout and autism spectrum disorder. The aim of the present study is to introduce the field of autism spectrum disorder in occupational groups, to assess the AQ10 as a screening mechanism, to discuss two conceptualisations and measures of burnout among clergy, and to identify the control variables that need to be taken into account when assessing connections between scores recorded on the AQ10 and individual differences in clergy burnout.

### Autism Spectrum Disorder in Occupational Groups

Autism Spectrum Disorder (ASD) is a neurodevelopmental condition characterised by pervasive difficulties in communication and social interaction occurring independently from intellectual functioning (Word Health Organization, 2022). Scholars have taken a slow but emerging interest in the presence of autistic traits among certain professional groups. Several studies lend credence to the intuition that ‘high-functioning’ people with an autistic condition often gravitate toward the STEM professions (Baron-Cohen et al., 2001, 2007; Turner et al., 2021). In particular, recent attention has been given to links between autistic traits and psychological distress such as depression and burnout among Japanese medical students and pharmacists (Higuchi et al., 2015, 2016, 2017; Watanabe et al., 2021; Watanabe & Akechi, 2023). Likewise, there is a growing interest in the wellbeing of autistic doctors in the UK (Doherty et al., 2021; Moore et al., 2020; Price et al., 2019), as well as the lived experiences of autistic legal professionals (McKinney, 2020; Ward, 2019), autistic educators (Kaupins et al., 2020; Martin, 2021; Wright & Kaupins, 2017), and autistic psychologists (Hawker et al., 2022). As yet clergy and religious professionals have not featured strongly in this stream of research.

### Introducing the AQ10 as a Screening Mechanism

The Autism Spectrum Quotient (AQ) was developed initially by Baron-Cohen et al. (2001) as a 50-item screening mechanism for identifying individuals for referral to professional assessment for diagnosis of autism spectrum disorders. In addition to its function as a clinical screening tool, the 50-item AQ has been used by researchers to examine its relationship to a variety of experiences such as schizophrenia (Lugnegard et al., 2015), disordered eating (Barnett et al., 2021), gender dysphoria (Heylens et al., 2018), and belief in God (Norenzayan et al., 2012). Allison et al. (2012) subsequently condensed this 50-item measure to a 10-item version which has been endorsed by the UK National Institute for Health and Care Excellence for screening ‘normal’ intelligence adults (National Institute for Clinical Excellence, 2021). The AQ10 identifies ten ‘red flags’ as potential indicators of ASD and proposes the cut-off point of ‘six or more’ red flags as the threshold for referral to professional assessment. Within the extant literatures this threshold has sometimes been mis-reported as ‘more than six’ red flags (see Waldren et al., 2021).

Although the validity of longer versions such as the AQ50 and AQ38 is supported by some studies (Booth et al., 2013; Hoekstra et al., 2008; Lau et al., 2013; Woodbury-Smith et al., 2005), other studies have questioned the predictive power of the shorter AQ28, AQ20, and AQ10 versions for diagnosing autism (Ashwood et al., 2016; Brugha et al., 2011; Sizoo et al., 2015). For example, Brugha et al. (2011) found that the AQ10 only moderately predicted ASD diagnosis within a large general population. Ashwood et al. (2016) found that the AQ10 ‘was not better than chance as a predictor of ASD diagnosis’ and cautioned that the measure may be picking up wider generalised anxiety disorder (GAD) and related pathologies.

In analyses of the AQ50 in non-clinical samples, two factors (Hoekstra et al., 2008) three factors (Austin, 2005; Hurst et al., 2007; Palmer et al., 2015), and four factors (Freeth et al., 2013; Stewart & Austin, 2009) have emerged. The psychometric performance of the AQ10 has also been variable. Taylor et al. (2020) reviewed the psychometric performance of the AQ10 in a large non-clinical sample of UK adults and found that the unidimensional model was a poor fit and that the instrument seemed to be measuring four different constructs. Lundin et al. (2019) conducted a similar study with Swedish adults and, although the unidimensionality was retained, two items concerning ‘attention to detail’ and ‘imagination’ did not fit well. In contrast, Jia et al. (2019) derived the AQ9 and found that two factors involving ‘social communication’ and ‘attention to detail’ worked better than the five-factor model in a sample of university students.

### Reconceptualising the AQ10 as a Diagnostic tool

Although Ashwood et al. (2016) did not find the AQ50 or the AQ10 to be good predictors of an autism diagnosis, they found that diagnosis of generalised anxiety disorder could predict high scores on the AQ10. Other studies have also highlighted the comorbid nature of the AQ10. For example, Mazzoli et al. (2022) found a significant decrease in the AQ scores of transgender people receiving hormones in parallel to decreased alexithymia and social anxiety after one year, suggesting that the AQ may have been detecting symptoms of anxiety rather than autism. Similarly, decreased depression was accompanied by decreased AQ scores in a study involving primary care patients (Ishizuka et al., 2022).

The analysis of Ashwood et al. (2016) may open the utility of the easily administered AQ10 to an additional application alongside its primary application of identifying referral to professional assessment for ASD. It is against this background that the present study sets out to explore the wider application of the AQ10 in the prediction of professional burnout and work-related psychological wellbeing among Anglican clergy. To advance this objective, consideration needs to be given to two issues: the conceptualisation and assessment of burnout among clergy, and the control variables that may need to be taken into account in exploring the connection between AQ10 scores and burnout scores.

### Assessing Burnout Among Clergy

The two conceptualisations of burnout most frequently employed among clergy are the three-factor model proposed by the Maslach Burnout Inventory (MBI; Maslach & Jackson, 1986) and the two-factor model proposed by the Francis Burnout Inventory (FBI; Francis et al., 2005). There are two fundamental differences between these two models. First, the MBI conceptualises burnout in terms of sequential progression across the three factors: emotional exhaustion leads to depersonalisation, and depersonalisation leads to lack of personal accomplishment. The FBI conceptualises burnout in terms of the classic balanced affect approach to wellbeing (Bradburn, 1969): positive affect and negative affect operate as partially independent systems within which positive affect can ameliorate the detrimental consequences of negative affect. Second, while the MBI was developed for generic use among people-facing professions, the FBI was developed specifically for use among clergy, drawing on work-related experiences specifically shaped by the clerical experience. The two components of the FBI are styled the Scale of Emotional Exhaustion in Ministry (SEEM) and the Satisfaction in Ministry Scale (SIMS).

The practical insight arising from the balanced affect model of clergy burnout is that, although it may be difficult to remove from clergy experience the factors that generate negative affect, good pastoral oversight can facilitate the development of factors that support positive affect. The validity of this theory has now been established in a series of studies among 744 clergy serving in The Presbyterian Church USA (Francis et al., 2011), 155 Catholic priests serving in Italy (Francis et al., 2017a), 95 Catholic priests and 61 Catholic religious sisters serving in Italy (Francis et al., 2017b), 658 Anglican clergy serving in England (Francis et al., 2017c), 358 Anglican clergy serving in Wales (Village et al., 2018), 90 Anglican clergy serving in England (Francis et al., 2019), 287 Catholic priests serving in Italy (Francis et al., 2021), and 803 Methodist ministers serving in Great Britain (Francis et al., 2023). The present study employs the FBI.

### Taking Control Variables into Account

The consensus emerging from earlier research employing either the MBI or the FBI among clergy is that individual differences in burnout may be mapped against both personal and personality factors (for review see Francis, 2018). Of the two key personal factors (sex and age), age is the more important. In survey studies of burnout among clergy, burnout scores are routinely found to be higher among younger clergy. Two theories have been advanced to account for this routine finding: an ageing effect is reflected in older clergy being better able to manage their work-related psychological wellbeing; a cohort effect is reflected in those clergy more susceptible to burnout dropping out at a younger age.

Of the range of personality factors included in survey studies of burnout among clergy, neuroticism or emotionality routinely emerges as the strongest predictor, followed by extraversion in the second place. Emotionality and extraversion are two aspects of personality that feature across diverse models of personality including the 16 Personality Factor model (Cattell et al., 1970), the Big Five Factor model (Costa & McCrae, 1985), the Eysenckian Three Major Dimensions model (Eysenck & Eysenck, 1991), and the augmented model of psychological type theory operationalised by the Francis Psychological Type and Emotional Temperament Scales (FPTETS; Village & Francis, 2023). The present study employs the FPTETS. The personal factor of sex differences remains important in light of the established association between sex and measures of emotionality (Francis, 1993).

### Research Questions

Against this background, the present study set out to explore the following sequence of four research questions. The first research question set out to explore the psychometric properties of the Autism Spectrum Quotient (AQ10) among clergy in terms of the internal consistency reliability in order to assess the utility of this instrument to be employed as a continuum measure of individual differences in predisposition to autistic tendencies among Anglican clergy.

The second research question set out to explore the proportion of Anglican clergy who answered each of the ten items on the AQ10 in the direction of recording a ‘red flag’ for predisposition to autism.

The third research question set out to explore the proportion of Anglican clergy who recorded six or more red flags and who on this criterion would be considered for referral for specialist diagnostic assessment.

The fourth research question set out to explore the association between scores recorded on the AQ10 and both emotional exhaustion in ministry and satisfaction in ministry after taking into account personal factors (age and sex) and personality factors (extraversion and emotionality).

## Method

### Procedure

Anglican clergy listed on the six diocesan websites within the Church in Wales as holding either a licence or permission to officiate were mailed a 16-page survey on ‘Resilience in Ministry’ and invited to return these surveys by FREEPOST to Dr V. John Payne. Responses were completely confidential and anonymous. A 25% response rate generated 244 completed surveys.

### Measures

*Personality factors* were assessed by the Francis Psychological Type and Emotional Temperament Scales (FPTETS; Village & Francis, 2023) developed from the instrument originally proposed by Francis (2005). This 50-item instrument comprises five sets of 10 forced-choice items related to emotionality and to each of the four components of psychological type: orientation (extraversion or introversion), perceiving process (sensing or intuition), judging process (thinking or feeling), and attitude toward the outer world (judging or perceiving). A number of studies have demonstrated this instrument to function well in church-related contexts. For example, Francis et al. (2008) reported alpha coefficients of 0.83 for the EI scale, 0.76 for the SN scale, 0.73 for the TF scale, and 0.79 for the JP scale. Participants were presented each pair of characteristics and asked to ‘check the box next to that characteristic that is closer to the real you, even if you feel both characteristics apply to you. Tick the characteristics that reflect the real you, even if other people see you differently.’

*Professional burnout* was assessed by the two scales proposed by the Francis Burnout Inventory (FBI: Francis et al., 2005). This 22-item instrument comprises the Scale of Emotional Exhaustion in Ministry (SEEM) and the Satisfaction in Ministry Scale (SIMS). Each item is assessed on a five-point scale: ranging from agree strongly (5) through not certain (3) to disagree strongly (1). Francis et al. (2005) reported alpha coefficients of 0.84 for SIMS and 0.84 for SEEM.

*Autism predisposition* was assessed by the *Autism Spectrum Quotient* (AQ10) proposed by Allison et al. (2012). This 10-item instrument comprises a single measure. For application in multivariate statistical analysis each item is assessed on a four point scale: ranging from definitely agree (4) to definitely disagree (1), with six reverse coded items. For ‘red flag’ screening, the agree and agree strongly responses (after recoding) are combined to count as a positive ‘red flag’ indicator. The scoring guidelines recommend that ‘individuals scoring 6 or above’ should be considered for referral for specialist diagnostic assessment (National Institute for Clinical Excellence, 2021).

### Participants

The present analyses are conducted among the clergy who identified as engaged in aspects of parish ministry and who fully completed all the measures used in these analyses. Of the 244 completed surveys 220 participants satisfied these criteria for inclusion. Among these 220 participants 148 were male and 72 female; 5 were under the age of forty, 15 were in their forties, 36 were in their fifties, 76 were in their sixties, 68 were in their seventies, and 20 were aged eighty or over; 87 were engaged in stipendiary ministry, 37 were engaged in self-supporting ministry, and 96 were retired.

### Data Analysis

The data were analysed by the SPSS statistical package employing the frequency, reliability, correlations, and regression routines.

## Results and Discussion

**Table 1:** Scale properties

	N items	Range			Alpha	Mean	SD
		Lo		H			
Scale of Emotional Exhaustion in Ministry	11	11	-	55	0.84	26.0	7.0
Satisfaction in Ministry Scale	11	11	-	55	0.81	43.2	4.6
FPTETS Extraversion Scale	10	0	-	10	0.84	3.9	3.0
FPTETS Emotionality Scale	10	0	-	10	0.76	2.9	2.3
Autism Spectrum Quotient (AQ10)	10	10	-	40	0.54	21.0	3.7

The first step in data analysis involved exploring the psychometric properties of the five measures employed in the present study in terms of the internal consistency reliability reported by the alpha coefficient (Cronbach, 1951) and the means and standard deviations. The data presented in Table 1 demonstrate that the Scale of Emotional Exhaustion in Ministry, the Satisfaction in Ministry Scale, the FPTETS Extraversion Scale, and the FPTETS Emotionality Scale all achieved satisfactory levels of internal consistency reliability. The Autism Spectrum Quotient (AQ10), however, failed to achieve an acceptable level of internal consistency reliability by generally recognised criteria as proposed by DeVellis (2003).

**Table 2:** AQ10: scale properties

	r	DA %	SA %	SD %	DD %
I often notice small sounds when others do not	− 0.15	20	36	21	23
I usually concentrate more on the whole picture, rather than the small details^*^	0.24	39	43	17	2
I find it easy to do more than one thing at once^*^	0.32	25	33	30	12
If there is an interruption, I can switch back to what I was doing very quickly^*^	0.39	30	43	25	2
I find it easy to ‘read between the lines’ when someone is talking to me^*^	0.51	28	51	18	3
I know how to tell if someone listening to me is getting bored^*^	0.41	40	53	6	1
When I’m reading a story I find it difficult to work out the characters’ intentions	0.16	3	28	43	26
I like to collect information about categories of things (e.g. types of car, types of bird, types of train, types of plant etc.)	0.12	8	24	29	39
I find it easy to work out what someone is thinking or feeling just by looking at their face^*^	0.31	12	47	35	6
I find it difficult to work out people’s intentions	0.30	4	27	57	12

Note: *r* = correlation between item and sum of other nine items

DA = Definitely Agree; SA = Slightly Agree; SD = Slightly Disagree; DD = Definitely Disagree

^*^ = item reverse coded to calculate *r*

*N* = 220

The second step in data analysis involved examining in greater detail the performance of the individual items within the AQ10 in terms of the correlations between the individual items and the sum of the other nine items. The data presented in Table 2 help to explain the low alpha coefficient achieved by this measure. In particular, the item ‘I often notice small sounds when others do not’ failed to covary in the expected direction with the other items.

The data in Table 2 also present the endorsement of the ten items in terms of the proportions of clergy who checked the definitely agree, slightly agree, slightly disagree, and definitely disagree responses. In terms of the four positively worded items, a red flag is achieved by checking definitely agree or slightly agree. By this criterion, nearly a third of the clergy scored a red flag on three items: I find it difficult to work out people’s intentions (31%); When I am reading a story I find it difficult to work out the characters’ intentions (31%); and I like to collect information about categories of things (32%). Over half of the clergy scored a red flag on the fourth of the positively worded items: I often notice small sounds when others do not (56%). In terms of the six negatively worded items, a red flag is achieved by checking slightly disagree or definitely disagree. By this criterion, two-fifths of the clergy scored a red flag on two items: I find it easy to do more than one thing at once (42%); and I find it easy to work out what someone is thinking or feeling just by looking at their face (41%). More than one fifth of the clergy scored a red flag on two items: If there is an interruption I can switch back to what I was doing very quickly (27%); and I find it easy to read between the lines when someone is talking to me (21%). On the remaining two items, a smaller proportion of clergy scored a red flag: I usually concentrate more on the whole picture, rather than the small details (19%); and I know how to tell if someone listening to me is getting bored (7%).

**Table 3:** Autism spectrum quotient (index 0 to 10)

Score	%
0	3.6
1	13.2
2	26.8
3	19.1
4	16.8
5	11.8
6	5.0
7	1.8
8	1.4
9	0.4
10	0.0

The third step in data analysis involved scanning the red flags. Table 3 demonstrates that 8.6% of the clergy recorded six or more red flags. This finding can be located alongside other recent surveys employing the AQ10 among non-clinical populations. Gollwitzer et al. (2019) reported on 6,595 participants spread across 104 countries who responded to an online social psychological skill quiz: 8.4% recorded six or more red flags. Lundin et al. (2019) reported on 44,722 participants in a public health survey collected by Statistics Sweden on behalf of the Stockholm County Council: overall 7.7% recorded six or more red flags, with variation among different groups of men between 6.8% and 14.9% and among different groups of women between 3.6% and 10.2%.

**Table 4:** Correlation matrix

	AQ9	SIMS	SEEM	EM	EX	Age
Sex	− 0.30^***^	− 0.01	− 0.10	− 0.06	0.12	− 0.17^*^
Age	0.17^*^	0.03	− 0.25^***^	− 0.15^*^	0.12	
Extraversion (EX)	− 0.27^***^	0.31^***^	− 0.25^***^	− 0.14^*^		
Emotionality (EM)	0.30^***^	− 0.29^***^	0.49^***^			
SEEM	0.27^***^	− 0.50^***^				
SIMS	− 0.43^***^					

Note: ^*^p < .05; ^**^p < .01; ^***^p < .001

Given the low internal consistency reliability (α = 0.54) recorded by the AQ10, and following the precedent of Jia et al. (2019), the worst performing item was dropped from the AQ10 to produce the AQ9 (α = 0.64). The fourth step in data analysis explored the bivariate correlations between scores recorded on the AQ9, personal factors (age and sex), personality factors (extraversion and emotionality), and professional burnout (emotional exhaustion in ministry and satisfaction in ministry). Three features of the data presented in Table 4 deserve commentary. First, scores recorded on the AQ9 were significantly related to personal factors, psychological factors, and burnout. Higher scores on the AQ9 were associated with older clergy and with male clergy. Higher scores on the AQ9 were associated with introversion and with emotionality. Higher scores on the AQ9 were associated with higher levels of emotional exhaustion in ministry and with lower levels of satisfaction in ministry. Second, older clergy reported lower levels of emotional exhaustion in ministry and lower levels of emotionality. This is consistent with other studies and is consistent with the theory that clergy who experience high levels of emotional exhaustion may drop out of ministry at a younger age and consequently no longer remain within the survey sample of clergy actively engaged in ministry (Francis, 2018). Third, not only are personality factors significantly correlated with AQ9 scores, they are also correlated significantly with both emotional exhaustion in ministry and satisfaction in ministry. These data confirm the importance of taking personality into account when exploring the connection between the AQ9 and burnout.

**Table 5:** Regression on satisfaction in ministry scale

	Model 1	Model 2	Model 3
*Personal factors*			
Sex	− 0.01	− 0.07	− 0.14^*^
Age	0.03	− 0.05	0.03
*Personality factors*			
Extraversion		0.28^***^	0.18^**^
Emotionality		− 0.26^***^	− 0.16^*^
*Autism Spectrum Disorders*			
AQ9			− 0.39^***^
R^2^	0.00	0.16	0.28
∆	0.00	0.16^***^	0.12^***^

Note: ^*^p < .05; ^**^p < .01; ^***^p < .001

**Table 6:** Regression on scale of emotional exhaustion in ministry

	Model 1	Model 2	Model 3
*Personal factors*			
Sex	− 0.15^*^	− 0.09	− 0.07
Age	− 0.27^***^	− 0.18^**^	− 0.21^***^
*Personality factors*			
Extraversion		− 0.16^**^	− 0.13^*^
Emotionality		0.43^***^	0.39^***^
*Autism Spectrum Disorders*			
AQ9			0.12^*^
R^2^	0.08	0.30	0.31
∆	0.08^***^	0.22^***^	0.01^*^

Note: ^*^p < .05; ^**^p < .01; ^***^p < .001

The final step in data analysis explores the associations between the AQ9 and both emotional exhaustion in ministry and satisfaction in ministry, with a stepwise regression model, entering personal factors (age and sex) in step one, psychological factors (extraversion and emotionality) in step two, and the AQ9 in step three. Table 5 demonstrates that age was not a significant predictor of individual differences in satisfaction in ministry, and that clergywomen recorded lower levels of satisfaction in ministry compared with clergymen. Both extraversion and emotionality were significant predictors of individual differences in satisfaction in ministry: higher levels of satisfaction in ministry were associated with stable extraversion. This finding is consistent with the broader finding within positive psychology that positive affect is higher among stable extraverts (Eysenck, 1983; Francis, 1999). After personal factors and psychological factors were taken into account, higher scores on the AQ9 were associated with significantly lower levels of satisfaction in ministry. Table 6 demonstrates that age (but not sex) is a significant predictor of individual differences in emotional exhaustion in ministry. This finding is consistent with previous research (Francis, 2018). Both extraversion and emotionality were significant predictors of individual differences in emotional exhaustion in ministry. This finding too, is consistent with previous research (Francis, 2018). After personal and psychological factors were taken into account, higher scores on the AQ9 were associated with significantly higher levels of emotional exhaustion in ministry.

### Limitations

There are two limitations with the present study that limit the generalisability of the two main findings in different ways. The first main finding is that screening with the AQ10 may be helpful in identifying clergy vulnerable to professional burnout and to poor work-related psychological wellbeing. This is a correlational finding so far established on just one sample of 220 Anglican clergy. This finding is, nonetheless, sufficiently intriguing to stimulate further replication studies among other groups of clergy. The second main finding is that 8.6% of the participants recorded six or more red flags on the AQ10, and so qualified for special diagnostic assessment. This is a finding based on a response rate of 25% and with 10% of the participants not providing full data. This finding now needs testing against a full population study. The third limitation with the present study is that it was not designed to test the validity of referrals recommended by the AQ10 among clergy for diagnostic assessment. Further replication and extension studies are now required in order both to test the predictive power of the AQ10 among clergy more fully and to follow up those who record six or more red flags with referral for specialist diagnostic assessment.

## Conclusion

This study set out to explore a sequence of four research questions employing the AQ10 among clergy alongside an established measure of professional burnout or poor work-related psychological wellbeing. These four research questions were addressed on data provided by 220 Anglican clergy serving in the Church in Wales and currently engaged in active parish ministry. The data lead to the following conclusions.

The first research question explored the psychometric properties of the Autism Spectrum Quotient (AQ10) among clergy in terms of internal consistency reliability. The emerging alpha coefficient of 0.54 failed to reach an acceptable level of internal consistency reliability as generally required for psychometric assessment tools (DeVellis, 2003). Closer examination of the items identified some items with particularly poor fit. This finding caveats the usefulness among clergy of employing scale scores computed on the AQ10 in correlational and regression analyses. As a consequence, and following an earlier precedent, the worst performing of the ten-items was dropped to produce the AQ9 that was then used subsequently in the correlational analysis and the regression models.

The second research question explored the proportion of Anglican clergy who answered each of the ten items on the AQ10 in the direction of recording a ‘red flag’ for predisposition to autism. In accordance with good practice in instruments of this kind, the individual items recorded a wide range of discrimination: with one item raising a red flag for 7% and another for 56% of the clergy. In other words, the individual items contributed differently to the diagnostic process.

The third research question explored the proportion of Anglican clergy who recorded six or more red flags and who on this criterion would be considered for specialist diagnostic referral. According to this criterion, 8.6% of the clergy participants would have been referred for specialist diagnostic referral. This percentage is roughly consistent with the findings from other studies conducted among non-clinical samples (Gollwitzer et al., 2019, Lundin, 2019), suggesting that rates for referral among clergy to clinical assessment are at levels expected in the general population.

The fourth research question conceptualised the AQ9 as a diagnostic tool with the additional capacity to identify wider generalised affective disorders, and then explored the association between scores recorded on the AQ9 and both emotional exhaustion in ministry and satisfaction in ministry, after controlling for pertinent personal and personality factors. Here, the data demonstrated that higher scores recorded on the AQ9 predicted significantly lower scores of satisfaction in ministry (*β* = − 0.39^***^), and significantly higher levels of emotional exhaustion in ministry (*β* = 0.12^*^). This finding is of both theoretical and practical interest within the balanced affect model of psychological wellbeing as operationalised by the Francis Burnout Inventory. Theoretical interest resides in the finding that the AQ9 is a much stronger predictor of scores in satisfaction in ministry (positive affect) than of scores in emotional exhaustion in ministry (negative affect) after personal and personality factors have been taken into account. This finding offers further validation for the basic thesis of the balanced affect model that positive affect and negative affect operate as partly independent systems.

Practical interest resides in the implications of this finding for the pastoral support of clergy. The first established insight from this model is that the deleterious consequence of negative affect can be mitigated by positive affect. The second established insight is that intervention strategies designed to promote clergy wellbeing may be more profitably targeted at enhancing positive affect than at trying to reduce the causes of negative affect. The new insight offered by the present study is that routine assessment with the AQ9 (conceptualised as a broad measure of generalised affective disorders) is able to identify those clergy most vulnerable to low levels of positive work-related affect. These may be the clergy most in need of appropriate intervention strategies and the most challenging to impact by such strategies.

Finally, the jury still remains out on the usefulness of the AQ10 for identifying clergy with ASD. While some, like Ashwood et al. (2016), may question the predictive power of the AQ10, the National Institute for Clinical Excellence (2021) retains clinical confidence in this measure. What is now needed is a systematic programme whereby clergy who record six or more red flags on the AQ10 are referred for specialist diagnostic assessment and the predictive power of the referral monitored.

## Data Availability

Data are available from the corresponding author upon reasonable request.
